# First-Pass Isolation as an Independent Predictor of Atrial Fibrillation Recurrence After Cryoballoon Ablation in Patients with Persistent Atrial Fibrillation

**DOI:** 10.3390/jcm14248914

**Published:** 2025-12-17

**Authors:** Seongjin Park, Hyo Jin Lee, Jiwon Kim, Juwon Kim, Ju Youn Kim, Seung-Jung Park, Kyoung-Min Park, Young Keun On

**Affiliations:** 1 Division of Cardiology, Department of Internal Medicine, Heart Vascular Stroke Institute, Samsung Medical Center, Sungkyunkwan University School of Medicine, Seoul 06351, Republic of Korea; ferryst@naver.com (S.P.); orthovics@gmail.com (S.-J.P.);; 2 Division of Cardiology, Department of Internal Medicine, Samsung Changwon Hospital, Sungkyunkwan University School of Medicine, Changwon 51353, Republic of Korea; hyozhin@naver.com; 3 Division of Cardiology, Department of Internal Medicine, Samsung Kangbuk Hospital, Sungkyunkwan University School of Medicine, Seoul 03181, Republic of Korea

**Keywords:** atrial fibrillation, persistent atrial fibrillation, cryoablation, first-pass isolation

## Abstract

**Background/Objectives**: Data on predictors of atrial fibrillation (AF) recurrence after cryoballoon pulmonary vein isolation (PVI) in persistent AF (PeAF) remain limited. We evaluated clinical, echocardiographic, and procedural parameters associated with recurrence. **Methods**: We retrospectively studied 192 PeAF patients who underwent cryoballoon PVI and had ≥6 months of follow-up. Recurrence was any atrial tachyarrhythmia > 30 s beyond a 3-month blanking period. Cox models assessed predictors, including number of veins with first-pass isolation (FPI), left atrial volume index (LAVI), hemoglobin, and prespecified covariates. **Results**: During a median follow-up of 670 days (interquartile range 425–944), recurrence occurred in 75 patients (39.1%). On multivariable analysis, reduced extent of FPI (<3 veins) independently predicted recurrence (HR 2.48, 95% CI 1.48–4.16; *p* = 0.001). In continuous analysis, each one-vein decrement in FPI was associated with a 58% higher hazard. Lower hemoglobin was also independently associated; each 1 g/dL decrease corresponded to a 29% higher hazard (HR 1.29, 95% CI 1.05–1.58; *p* = 0.015). Male sex showed approximately a twofold higher recurrence risk than female sex (HR 2.23, 95% CI 1.06–4.68; *p* = 0.034). In PeAF patients treated with cryoballoon PVI, intraprocedural FPI extent was a strong independent predictor, outperforming anatomical remodeling after adjustment. Achieving FPI in fewer than three pulmonary veins predicted higher recurrence. Hemoglobin provides an accessible systemic risk marker, while male sex identifies a higher-risk subgroup. **Conclusions**: In PeAF patients, the extent of FPI in cryoballoon PVI is a strong independent predictor of outcome, and maximizing the number of veins with FPI could be a pragmatic procedural goal.

## 1. Introduction

Pulmonary vein isolation (PVI) is established as the cornerstone lesion set for catheter ablation of atrial fibrillation (AF), and the 2024 European Society of Cardiology (ESC) guidelines reaffirm PVI as the only ablation strategy with a Class I recommendation for both paroxysmal and persistent AF in patients who are resistant or intolerant to antiarrhythmic drugs [1]. Cryoballoon ablation (CBA) is widely used to achieve PVI and is particularly effective in patients with paroxysmal AF [2]. However, outcomes for persistent AF (PeAF) remain inferior, likely due to advanced atrial remodeling and fibrosis, complicating procedural success and long-term rhythm control [3,4]. Previous studies reported variable recurrence rates after CBA for PeAF, emphasizing the clinical need for effective risk stratification and prediction of outcomes in this patient subset [5,6].

Several studies have identified clinical and anatomical predictors of recurrence, such as left atrial size, AF duration, and the interval from AF diagnosis to ablation [3,5]. Nevertheless, the identification of modifiable, intraprocedural predictors that can be directly modified during ablation remains relatively unexplored. Defining such procedural factors may facilitate improved patient selection, procedural optimization, and ultimately better clinical outcomes.

While PVI is the fundamental goal of CBA, procedural features—such as the extent of first-pass isolation (FPI) and the applied lesion set—have also been considered as potential predictors of recurrence [7,8,9]. In addition, left atrial structural parameters and systemic factors may contribute to arrhythmia recurrence in this heterogeneous population.

Therefore, this study aimed to identify independent predictors of AF recurrence among PeAF patients who underwent CBA with successful PVI. We comprehensively evaluated clinical, echocardiographic, and procedural parameters—including FPI, left atrial volume index (LAVI), and hemoglobin levels—to inform individualized risk stratification and guide procedural decision-making.

## 2. Materials and Methods

### 2.1. Study Population

**Figure 1:**
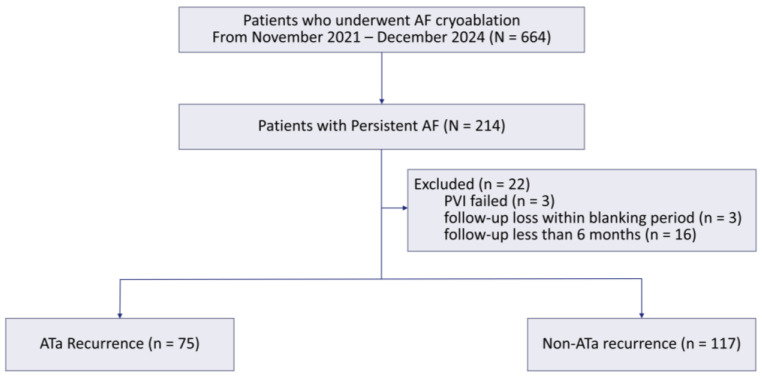
Study flow. Flow diagram of patient selection and study enrollment. Of 664 patients who underwent AF cryoablation between November 2021 and December 2024, 214 had persistent AF. The final analysis included 192 patients after excluding 3 patients with failed PVI, 3 patients who were lost to follow-up during the blanking period, and 16 patients who had less than 6 months of follow-up. AF, atrial fibrillation; PVI, pulmonary vein isolation; Ata, atrial tachyarrhythmia.

We retrospectively analyzed patients with PeAF who underwent CBA at Samsung Medical Center from November 2021 to December 2024. Among 214 PeAF patients treated throughout this period, we excluded those with failed PVI (*n* = 3), loss to follow-up within the blanking period (*n* = 3), and follow-up <6 months (*n* = 16), yielding a final analytic cohort of 192 patients with ≥6-month of total clinical follow-up from the index ablation to the last visit (Figure 1). To reduce era-related confounding and under-ascertainment of events in recently treated patients, we required at least 6 months of total follow-up, which ensured a minimum of 3 months of observation beyond the 3-month blanking period for all included patients. This study was approved by the Institutional Review Board of Samsung Medical Center, South Korea (IRB No. 2025-04-104).

### 2.2. Ablation Procedure

Procedures were performed under conscious sedation using intravenous midazolam and fentanyl. Sedation level was continuously monitored, and additional doses were administered as needed. If adequate sedation was not achieved, propofol was administered as a supplemental agent. Bilateral femoral venous access was obtained. A duodecapolar electrode catheter was positioned in the coronary sinus and right atrium, and a deflectable His-RV electrode catheter was used for His bundle and right ventricular recordings. Intracardiac echocardiography (ICE; ViewFlex™, Abbott, Abbott Park, IL, USA; or AcuNav™, Biosense Webster, Inc., Irvine, CA, USA) was introduced via the femoral vein to guide transseptal access and monitor catheter positioning.

Transseptal puncture was performed under ICE guidance using a Brockenbrough needle (Abbott, Abbott Park, IL, USA), and the delivery sheath was advanced into the left atrium. After transseptal puncture, intravenous heparin was administered to initiate anticoagulation. Activated clotting time (ACT) was monitored every 30 min during the procedure, and additional heparin was administered as needed to maintain an ACT > 300 s. At the end of the procedure, protamine sulfate was administered to reverse the effects of heparin.

CBA was performed using either the Arctic Front Advance Pro system (Medtronic, Dublin, Ireland) or the POLARx system (Boston Scientific, Marlborough, MA, USA) at the operator’s discretion. A 28 mm cryoballoon catheter was used in all cases. Each pulmonary vein was targeted with a freeze cycle of 3 to 4 min. Balloon positioning and vein occlusion were assessed using ICE and contrast injection as needed. PVI was confirmed with a circular mapping catheter (Achieve™, Medtronic, Dublin, Ireland; or POLARMAP™, Boston Scientific, Marlborough, MA, USA). FPI was defined as electrical isolation achieved with the initial cryoablation application without additional freezes. If FPI was not achieved, additional cryoablation applications were performed to ensure complete isolation of all four pulmonary veins. Additional ablation lesions, such as left atrial roof or superior vena cava isolation, were performed at the discretion of the operator.

### 2.3. Data Collection and Definition

Baseline demographics, clinical characteristics, echocardiographic parameters, and procedural data were collected from electronic medical records. Procedural variables included balloon type, total freeze time, the presence of additional ablation lesions, and the number of pulmonary veins achieving FPI. All patients were followed in the outpatient clinic with electrocardiogram (ECG), Holter monitoring, implantable loop recorder (ILR) interrogation, or smart device analysis, depending on physician preference. AF recurrence was defined as any documented atrial tachyarrhythmia (AF, atrial flutter, or atrial tachycardia) lasting more than 30 s, detected by any available monitoring modality beyond a 3-month blanking period. All device-detected episodes (ILR or wearable tracings) were reviewed and confirmed by an electrophysiologist.

### 2.4. Statistical Analysis

Continuous variables were expressed as mean ± standard deviation, and categorical variables were presented as frequencies and percentages. Comparisons between recurrence and non-recurrence groups were made using Student’s *t*-test or chi-square test, as appropriate. Univariate and multivariate Cox proportional hazards regression analyses were performed to identify independent predictors of AF recurrence. Variables with *p* < 0.1 in univariate analysis were entered into the multivariate model, along with clinically relevant factors (age, sex). Cutoffs for continuous variables were identified using the maximally selected rank statistics method (R package: maxstat, version 2.4). Kaplan–Meier survival curves were generated for recurrence-free survival comparisons. All statistical analyses were performed using SPSS version 26.0 (SPSS-PC, Chicago, IL, USA) and R version 4.4.3 (R Foundation for Statistical Computing, Vienna, Austria). *p*-values < 0.05 were considered statistically significant.

## 3. Results

### 3.1. Study Population and Baseline Characteristics

A total of 192 patients with PeAF who underwent successful cryoballoon PVI and had ≥6 months of follow-up were included in the analysis. The mean age of the cohort was 59.7 ± 10.2 years, and 81.3% were male. The median diagnosis-to-ablation interval was 3.3 ± 3.2 years. Hypertension (43.8%), diabetes mellitus (18.2%), and ischemic heart disease (16.1%) were the most common comorbidities, while 15.6% of patients had heart failure. The mean left ventricular ejection fraction (LVEF) was 58.9 ± 8.3%, and the mean left atrial volume index (LAVI) was 44.9 ± 13.6 mL/m^2^. The mean hemoglobin and N-terminal pro-B-type natriuretic peptide (NT-proBNP) levels were 14.0 ± 1.4 g/dL and 615.0 ± 840.6 pg/mL. Both cryoballoon platforms were used during the study period: Arctic Front Advance Pro in 133 patients (69.3%) and POLARx in 59 patients (30.7%) (Table 1).

### 3.2. Recurrence and Procedural Outcomes

During a median follow-up of 670 days (interquartile range 425–944 days; mean 698 ± 303 days), atrial tachyarrhythmia recurrence occurred in 75 patients (39.1%). Baseline and procedural characteristics according to recurrence status are summarized (Table 2). The recurrence group showed fewer pulmonary veins achieving FPI (2.6 ± 1.1 vs. 3.0 ± 1.0, *p* = 0.007) and lower hemoglobin levels (13.8 ± 1.4 vs. 14.2 ± 1.3 g/dL, *p* = 0.041) compared with the non-recurrence group. LAVI was numerically higher without statistical significance in the recurrence group (47.0 ± 15.5 vs. 43.5 ± 12.1 mL/m^2^; *p* = 0.104). Other baseline, echocardiographic, and procedural parameters were comparable between the two groups. Acute procedural complications were infrequent. Transient phrenic nerve palsy occurred in two patients, and major vascular complications, including arteriovenous fistula and pseudoaneurysm, occurred in two patients, while no cases of cardiac tamponade, stroke, or transient ischemic attack were observed during or immediately after the procedures.

### 3.3. Independent Predictors of Recurrence

**Figure 2:**
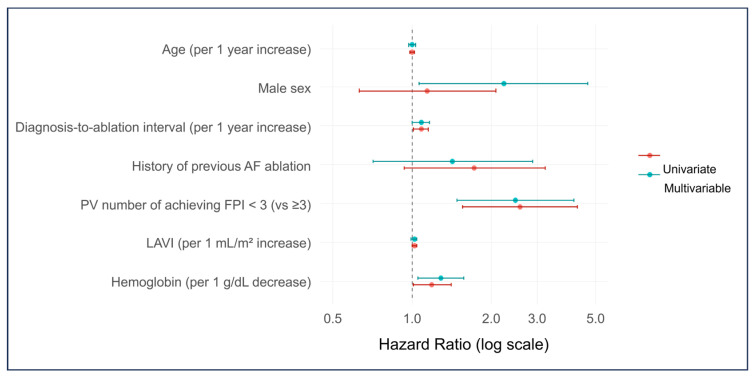
Univariable and multivariable predictors of atrial tachyarrhythmia recurrence. Forest plot showing hazard ratios with 95% confidence intervals for atrial tachyarrhythmia recurrence after cryoballoon ablation. Seven clinically relevant covariates were included in the multivariable model (age, male sex, diagnosis-to-ablation interval, history of previous AF ablation, PV number of achieving FPI < 3, LAVI, and hemoglobin). Red markers indicate univariable analyses, and blue markers indicate multivariable analyses. AF, atrial fibrillation; PV, pulmonary vein; FPI, first-pass isolation; LAVI, left atrial volume index.

**Figure 3:**
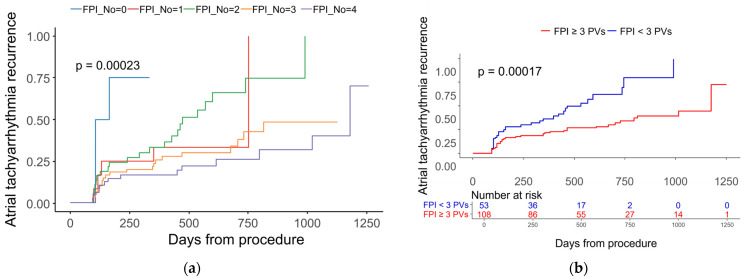
Association between the extent of first-pass isolation and atrial tachyarrhythmia recurrence. (**a**) Kaplan–Meier curves for time to first atrial tachyarrhythmia recurrence stratified by the number of pulmonary veins achieving FPI at the index procedure (0, 1, 2, 3, or 4 veins); (**b**) Kaplan–Meier curves comparing patients with FPI achieved in ≥3 versus <3 pulmonary veins. FPI, first-pass isolation.

**Figure 4:**
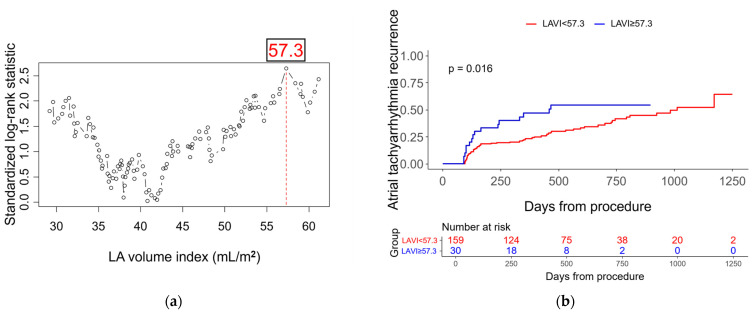
Optimal cutoff of left atrial volume index for predicting atrial fibrillation recurrence and corresponding Kaplan–Meier curves. (**a**) Identification of the optimal LAVI cutoff value (57.3 mL/m^2^) using maximally selected log-rank statistics; (**b**) Kaplan–Meier curves for atrial tachyarrhythmia recurrence according to the LAVI cutoff.

Univariate and multivariate Cox proportional hazards analyses were performed to identify independent predictors of AF recurrence (Table 3 and Figure 2). In the multivariate model, achieving FPI in fewer than three pulmonary veins was independently associated with a higher risk of recurrence (HR 2.48, 95% CI 1.48–4.16; *p* = 0.001). Kaplan–Meier curves showed a graded separation across FPI counts (0–4) (Figure 3a), and a pragmatic dichotomization also demonstrated significantly lower recurrence in patients with FPI achieved in ≥3 pulmonary veins compared with <3 (Figure 3b; log-rank *p* < 0.001). Consistently, in a continuous-scale analysis, each one-vein decrement in the number of veins achieving FPI was associated with a 58% higher hazard of recurrence (HR 1.58, 95% CI 1.25–2.00; *p* < 0.001; univariate). Lower hemoglobin independently predicted recurrence (per 1 g/dL decrease: HR 1.29, 95% CI 1.05–1.58; *p* = 0.015), and male sex showed an independent association (HR 2.23, 95% CI 1.06–4.68; *p* = 0.034). LAVI (per 1 mL/m^2^) was not retained in the multivariable model (*p* = 0.163). For descriptive purposes, a maximally selected rank statistics identified an optimal cutoff of 57.3 mL/m^2^; Kaplan–Meier analysis showed significantly higher recurrence among patients with LAVI ≥ 57.3 mL/m^2^ compared with <57.3 mL/m^2^ (Figure 4). Lesion set was not associated with recurrence (Table 3 and Supplementary Figure S1). The diagnosis-to-ablation interval demonstrated a borderline association, with a hazard ratio of 1.08 per year (95% CI 1.00–1.16; *p* = 0.063).

## 4. Discussion

CBA has become an increasingly utilized strategy for rhythm control in patients with PeAF. While PVI remains the cornerstone of ablation therapy for AF, outcomes in PeAF patients are generally less favorable than in paroxysmal AF, likely due to more advanced atrial remodeling and substrate heterogeneity [2,10,11]. And predictors of long-term success in PeAF remain not fully defined. Several prior studies have explored clinical, anatomical, and procedural factors influencing recurrence, including atrial volume, fibrosis burden, and procedural quality markers such as the extent of FPI [12,13,14,15]. More recently, ablation studies have also incorporated patient-reported outcomes such as quality of life, which are increasingly recognized as important for shared decision-making in AF care [16]. However, data remain limited regarding optimal procedural parameters and clinical predictors, specifically in PeAF cohorts undergoing CBA.

The present study is notable for identifying key clinical and procedural factors—reduced extent of FPI (fewer than three pulmonary veins), lower hemoglobin level, and male sex—that independently predicted arrhythmia recurrence in a relatively large PeAF cohort. Importantly, the use of two contemporary cryoballoon platforms (Medtronic and Boston) enhances the generalizability of our findings.

Intra-procedural FPI has emerged as a practical marker of lesion quality and durability. In large radiofrequency ablation (RFA) series, absence of FPI has been tied to higher rates of acute PV reconnection and worse long-term outcomes; for example, in a 446-patient study, veins with first-pass PVI still showed reconnection in 22% during waiting/adenosine triphosphate testing, but patients without first-pass PVI had significantly lower 2-year freedom from AF (75% vs. 59%; log-rank *p* = 0.032), underscoring both the value and the limits of FPI as a surrogate for durable block [7]. Evidence specific to PeAF further highlights that the extent of FPI (how many veins/sides achieve first-pass) matters prognostically. In a contemporary PeAF cohort, bilateral or at least ipsilateral FPI was associated with higher freedom from atrial tachyarrhythmias over ≈22 months compared with no FPI (bilateral 75.1%, ipsilateral 70.7% vs. neither 57.9%), and ipsilateral FPI remained an independent predictor on multivariable analysis [17]. From a procedural standpoint, FPI integrates several modifiable factors that influence lesion quality. Real-world registry data (REAL-AF, *n* = 2671) show FPI correlates with effective lesion formation and long-term freedom from recurrence, and identify modifiable predictors—e.g., higher posterior contact force, 50 W power, optimized ventilation, and sheath support—associated with improved odds of achieving FPI [8]. Although most FPI-prognostic literature comes from RFA cohorts, the pathophysiologic rationale extends to CBA, where complete PV occlusion and stable balloon–tissue contact are prerequisites for immediate block. Contemporary cryoballoon protocols explicitly verify occlusion with contrast injection as a quality step, reinforcing FPI (or immediate entrance/exit block) as a meaningful composite marker of contact/occlusion success [18]. In our PeAF cryoballoon cohort, the extent of FPI was independently associated with outcomes: patients with fewer than three pulmonary veins achieving FPI had a significantly higher hazard of recurrence (multivariable HR 2.48, 95% CI 1.48–4.16; *p* = 0.001). On a continuous scale, each one-vein decrement in the number of veins with FPI related to a 59% higher hazard in univariate analysis (HR 1.59, 95% CI 1.25–2.00; *p* < 0.001). In the era of high-power short-duration and very high-power short-duration RF and pentaspline pulsed field ablation, which achieve very high first-pass isolation rates and consistent acute PVI durability compared with conventional RF and some cryoballoon series [19], variability in FPI achievement—and thus the incremental prognostic value of FPI—may be attenuated. By contrast, in cryoballoon PVI, where multi-vein FPI is not uniformly achieved, our data indicate that FPI extent remains an important determinant of outcome in PeAF. Overall, vein-by-vein FPI provides a practical intraprocedural quality signal; maximizing the number of veins with FPI may help improve durability.

Beyond per-vein quality metrics such as FPI, whether empiric substrate modification should be added in PeAF remains unsettled. Large randomized RFA-era trials—most notably STAR-AF II—showed no reduction in recurrence when linear lesions or CFAE were added to PVI in PeAF [20]. The multicenter CAPLA trial likewise found no improvement with posterior wall isolation (PWI) added to wide-antral PVI at 12 months [21]. In contrast, a small, cryoballoon-only RCT reported improved outcomes with adjunctive PWI (24% vs. 46% recurrence; adjusted HR ≈ 0.26), although complete PWI was achieved in only 62% and the sample size was modest [9]. In our cohort, operator-selected extra lesions—predominantly roof or SVC isolation rather than systematic PWI—were not independently associated with lower recurrence, potentially reflecting confounding by indication and variable lesion durability. Taken together, these data support prioritizing attempts to achieve multi-vein FPI in unselected PeAF, while reserving lesion expansion for anatomically or electrophysiologically targeted scenarios and for prospective evaluation.

Lower hemoglobin was independently associated with recurrence after adjustment. This observation is consistent with prior reports linking anemia and low hemoglobin levels to adverse outcomes in AF, in which chronic systemic inflammation, iron dysregulation, and reduced oxygen delivery are proposed to accelerate atrial remodeling and fibrosis [22,23,24]. A data-driven cutoff of 13.8 g/dL was identified but does not correspond to a conventional clinical anemia threshold and should therefore be regarded as exploratory rather than a validated treatment target. Accordingly, we regard hemoglobin as a simple adjunctive risk marker, and further validation is required.

Sex-based differences in atrial remodeling and electrophysiological properties have been increasingly recognized in AF management. In our analysis, male sex was independently associated with higher recurrence after CBA (adjusted HR 2.23, 95% CI 1.06–4.68; *p* = 0.034), although this association was not significant in univariate comparisons. Previous studies, including those by Masuda et al. and Duarte et al., have suggested that men may exhibit greater atrial fibrosis burden and adverse substrate features, contributing to higher recurrence rates following ablation [25,26]. Experimental data have also indicated possible intrinsic electrophysiological differences, such as enhanced Na^+^/Ca^2+^ exchanger activity in males, which may predispose to arrhythmogenesis [27]. However, clinical studies have reported inconsistent findings regarding sex-related differences in ablation outcomes, highlighting the complex interplay between biological sex, atrial substrate, and procedural factors [28,29]. In line with these observations, our results support the potential relevance of sex in risk stratification for PeAF ablation, though further research is needed to clarify its role.

Left atrial remodeling plays a central role in the persistence of AF and its resistance to catheter ablation. Among remodeling markers, the LAVI has been consistently reported as a predictor of AF recurrence across modalities. More recently, the broader concept of atrial cardiomyopathy has highlighted that functional and substrate-based indices, particularly left atrial reservoir and conduit strain, may outperform simple volumetric markers such as LAVI in predicting AF recurrence after ablation [30]. In addition, mechanistic hallmarks of atrial cardiomyopathy, including fibrosis on late gadolinium enhancement cardiac magnetic resonance imaging and electroanatomic low-voltage substrate mapping, provide complementary information on the left atrial substrate and are increasingly incorporated into risk stratification [31,32,33]. In our cohort, LAVI did not retain independent significance once the extent of FPI was included in the model. Nevertheless, Kaplan–Meier analysis using a data-driven cutoff (57.3 mL/m^2^) demonstrated higher recurrence in patients with enlarged atria, reinforcing the prognostic relevance of structural remodeling. In addition, the curve of the standardized log-rank statistic across candidate LAVI cutoffs showed a U-shaped pattern, with higher values at both low and high LAVI ranges (Figure 4a). This exploratory finding raises the possibility that not only markedly enlarged but also very small atria may be prone to recurrence, potentially because maneuvering a large-bore steerable sheath and cryoballoon within a small left atrium can lead to suboptimal balloon engagement at the pulmonary vein antra. This observation should be regarded as hypothesis-generating and warrants validation through formal non-linear modeling in larger cohorts.

Beyond baseline and intraprocedural predictors, post-ablation management may also influence long-term rhythm outcomes. Structured cardiac rehabilitation programs for patients with AF have been reported to improve functional capacity, support weight control, and optimize risk factor management, and have been associated with lower recurrence rates after catheter ablation [34]. Future studies integrating procedural endpoints such as FPI with rehabilitation-based risk modification may help define more comprehensive strategies to reduce recurrence.

## 5. Limitations

Several limitations of this study should be acknowledged. First, the retrospective design introduces the possibility of selection and information bias. Second, post-ablation rhythm monitoring was not standardized and was performed at the discretion of the treating physicians, which may have influenced the sensitivity of recurrence detection, particularly for asymptomatic episodes. Furthermore, emerging artificial intelligence tools applied to ILR and wearable ECG data may further enhance automated detection of atrial arrhythmias and individualized risk stratification. Third, although follow-up duration was generally adequate, patients without recurrence tended to have longer follow-up durations compared with those with recurrence, potentially introducing healthcare engagement bias. Fourth, the generalizability of our findings may be limited. Our cohort was derived from a single tertiary center with specific expertise in CBA, and a proportion of patients had a history of prior AF ablation. While this variable was included and adjusted for in the multivariable analysis, the presence of both de novo and redo cases and the dependence of FPI success on operator proficiency, experience with cryoballoon manipulation, and center-specific procedural protocols may have influenced recurrence rates and the observed FPI thresholds. Future studies focusing exclusively on first-time ablation cases and involving multiple centers may provide a clearer understanding of predictors in this setting. Finally, FPI was assessed intraprocedurally as an acute endpoint; the durability of PVI was not systematically adjudicated (e.g., with adenosine testing or remapping), which may have led to underdetection of latent reconnection. Larger, prospective studies are warranted to validate our findings and further investigate the impact of these predictors in PeAF patients undergoing CBA.

## 6. Conclusions

In this single-center cohort of patients with PeAF undergoing CBA, the extent of FPI emerged as a strong independent procedural predictor of outcome: achieving FPI in fewer than three pulmonary veins independently predicted higher arrhythmia recurrence, alongside lower hemoglobin and male sex. These findings emphasize that procedural quality (multi-vein FPI) and simple clinical markers can refine risk stratification beyond traditional structural parameters in PeAF. Prospective studies are warranted to validate these predictors and to determine whether intraprocedural strategies that increase multi-vein FPI translate into more durable PVI and improved clinical outcomes.

## Figures and Tables

**Table 1:** Baseline clinical and echocardiographic characteristics.

	Total
	*N* = 192
Age, y	59.7 ± 10.2
Sex (male)	156 (81.3)
BMI (kg/m^2^)	25.6 ± 3.5
CHA^2^DS^2^-VASc score	1.6 ± 1.3
Diagnosis-to-ablation interval (years)	3.3 ± 3.2
Platform	
PolarX (Boston)	59 (30.7)
Arctic Front Advance Pro (Medtronic)	133 (69.3)
Comorbidities	
Congestive heart failure	30 (15.6)
Hypertension	84 (43.8)
Diabetes mellitus	35 (18.2)
Ischemic heart disease	31 (16.1)
Chronic kidney disease	11 (5.7)
Echocardiographic parameters	
LV ejection fraction (%)	58.9 ± 8.3
LA volume index (mL/m^2^)	44.9 ± 13.6
E/e′ ratio	8.9 ± 3.4
Laboratory findings	
Hemoglobin (g/dL)	14.0 ± 1.4
Estimated GFR (mL/min/1.73 m^2^)	85.8 ± 15.6
NT-proBNP (pg/mL)	615.0 ± 840.6

Values are presented as means ± standard deviations or numbers (percentages). BMI, body mass index; LV, left ventricle; LA, left atrium; GFR, glomerular filtration rate; NT-proBNP, N-terminal pro-B-type natriuretic peptide.

**Table 2:** Comparison between recurrence and non-recurrence groups.

	Non-Recurrence	Recurrence	*p* Value
	*N* = 117 (60.9)	*N* = 75 (39.1)	
Age, y	60.0 ± 10.2	59.3 ± 10.4	0.637
Sex (male)	94 (80.3)	62 (82.7)	0.710
BMI (kg/m^2^)	25.7 ± 3.1	25.6 ± 4.1	* 0.833
CHA2DS2-VASc score	1.5 ± 1.3	1.6 ± 1.2	0.626
Comorbidities			
Congestive heart failure	15 (12.8)	15 (20.0)	0.222
Hypertension	48 (41.0)	36 (48.0)	0.373
Diabetes mellitus	21 (17.9)	14 (18.7)	1.000
Ischemic heart disease	17 (14.5)	14 (18.7)	0.547
Chronic kidney disease	6 (5.1)	5 (6.7)	* 0.754
Diagnosis-to-ablation interval (years)	3.0 ± 2.6	3.7 ± 4.0	* 0.176
Follow-up duration (days)	631 ± 285	803 ± 304	<0.001
AF-free duration (days)	631 ± 285	320 ± 268	<0.001
History of Previous AF ablation	12 (10.3)	12 (16.0)	0.268
Lesion set			0.595
PVI only	24 (20.5)	18 (24.0)	
PVI with additional lesions (e.g., SVC or roof)	93 (79.5)	57 (76.0)	
Procedure time (min)	88.2 ± 21.7	92.0 ± 22.0	0.252
Ablation time (min)	57.2 ± 14.9	60.8 ± 16.5	0.126
Fluoroscopic time (min)	22.3 ± 10.1	22.6 ± 8.8	0.866
PV number of achieving FPI	3.0 ± 1.0	2.6 ± 1.1	0.007
Echocardiographic parameters			
LV ejection fraction (%)	59.3 ± 8.3	58.4 ± 8.2	0.447
LA volume index (mL/m^2^)	43.5 ± 12.1	47.0 ± 15.5	* 0.104
E/e′ ratio	8.8 ± 3.1	9.2 ± 3.8	0.415
Laboratory findings			
Hemoglobin (g/dL)	14.2 ± 1.3	13.8 ± 1.4	0.041
Estimated GFR (mL/min/1.73 m^2^)	86.0 ± 15.0	85.4 ± 16.5	0.789
NT-proBNP (pg/mL)	555.5 ± 535.9	709.3 ± 1176.0	0.393

Values are presented as means ± standard deviations for continuous variables, and numbers (percentages) for categorical variables. Groups comparisons were performed using Student’s *t*-test or chi-square test. * Alternative tests were applied when assumptions for the primary tests were not met (e.g., Welch’s *t*-test for unequal variances; exact tests for small expected cell counts). AF, atrial fibrillation; BMI, body mass index; SVC, superior vena cava; PV, pulmonary vein; FPI, first-pass isolation; LV, left ventricle; LA, left atrium; GFR, glomerular filtration rate; NT-proBNP, N-terminal pro-B-type natriuretic peptide.

**Table 3:** Independent predictors for AF recurrence among persistent AF patients who underwent cryoablation.

	Univariate Analysis		* Multivariate Analysis	
	HR (95% CI)	*p* Value	HR (95% CI)	*p* Value
Age, per 1 year increase	1.00 (0.98–1.02)	0.948	1.00 (0.97–1.03)	0.880
Male	1.14 (0.63–2.08)	0.664	2.23 (1.06–4.68)	0.034
BMI, per 1 kg/m^2^ increase	0.98 (0.92–1.05)	0.571		
CHA2DS2-VASc score, per 1 score increase	1.03 (0.86–1.23)	0.737		
Comorbidities				
Congestive heart failure	1.41 (0.80–2.48)	0.241		
Hypertension	1.14 (0.73–1.80)	0.566		
Diabetes mellitus	1.03 (0.58–1.85)	0.917		
Ischemic heart disease	1.17 (0.65–2.10)	0.594		
Chronic kidney disease	1.60 (0.64–3.98)	0.312		
Diagnosis-to-ablation interval, per 1-year increase	1.08 (1.01–1.15)	0.035	1.08 (1.00–1.16)	0.063
History of previous AF ablation	1.72 (0.93–3.21)	0.086	1.43 (0.71–2.88)	0.317
General anesthesia	1.05 (0.58–1.92)	0.869		
Lesion				
PVI only	1.28 (0.75–2.17)	0.365		
PVI with additional lesions (e.g., SVC or roof)	0.78 (0.46–1.33)			
PV number of FPI < 3	2.58 (1.56–4.28)	<0.001	2.48 (1.48–4.16)	0.001
PV number of FPI, per 1 decrease	1.58 (1.25–2.00)	<0.001		
Echocardiographic parameters				
LV ejection fraction, per 1% increase	0.99 (0.97–1.02)	0.646		
LA volume index, per 1 mL/m^2^ increase	1.02 (1.00–1.04)	0.014	1.02 (0.99–1.04)	0.163
LA volume index ≥ 57.3 mL/m^2^	1.96 (1.12–3.44)	0.018		
E of e′, per 1 increase	1.04 (0.98–1.12)	0.212		
Hemoglobin, per 1.0 g/dL decrease	1.19 (1.01–1.41)	0.035	1.29 (1.05–1.58)	0.015
Hemoglobin < 13.8 g/dL	1.68 (1.06–2.68)	0.029		

* Multivariate Cox regression was adjusted for age, sex, history of previous AF ablation, diagnosis-to-ablation interval, PV number of FPI < 3, LA volume index, and hemoglobin. Univariate and multivariate HRs with 95% CIs are shown. AF, atrial fibrillation; BMI, body mass index; HR, hazard ratio; CI = confidence interval; SVC, superior vena cava; PV, pulmonary vein; PVI, PV isolation; FPI, first-pass isolation; LV, left ventricle; LA, left atrium.

## Data Availability

The datasets generated and/or analyzed during the current study are available from the corresponding author on reasonable request. The data are not publicly available due to institutional and ethical restrictions.

## Supplementary Materials

The following supporting information can be downloaded at: https://www.mdpi.com/article/10.3390/jcm14248914/s1, Figure S1: Kaplan–Meier curves according to lesion sets.

## Abbreviations

The following abbreviations are used in this manuscript:

AF	Atrial fibrillation
PVI	Pulmonary vein isolation
PeAF	Persistent AF
FPI	First-pass isolation
LAVI	Left atrial volume index
NT-proBNP	N-terminal pro-B-type natriuretic peptide
HR	Hazard Ratio
CBA	Cryoballoon ablation
ICE	Intracardiac echocardiography
ACT	Activated clotting time
ILR	Implantable loop recorder
RFA	Radiofrequency ablation
PWI	Posterior wall isolation
